# Point-of-Care Ultrasonography in a Pulmonary Hypertension Clinic: A Randomized Pilot Study

**DOI:** 10.3390/jcm12051752

**Published:** 2023-02-22

**Authors:** Avital Avriel, Anat Bar Lavie Shay, Anat Hershko Klement, Jonathan Taylor, David Shamia, Gal Tsaban, Mahmoud Abu-Shakra, John Granton, Lior Fuchs

**Affiliations:** 1 Assuta Medical Center, Ha-Refu’a St 7, Ashdod 7747629, Israel; 2 Faculty of Health Sciences, Ben-Gurion University, Beer-Sheva 8410501, Israel; 3 Department of Obstetrics and Gynecology, Hadassah Mount Scopus, Jerusalem 9765422, Israel; 4 Division of Pulmonary, Critical Care, and Sleep Medicine, Mount Sinai Hospital, Icahn School of Medicine at Mount Sinai, New York, NY 10029, USA; 5 Division of Medicine, Soroka University Medical Center, Ben-Gurion University of the Negev, Beer-Sheva 8410101, Israel; 6 Department of Medicine, University of Toronto, Toronto, ON M5S 1A8, Canada; 7 Respirology, Pulmonary Hypertension Program, University Health Network, Toronto, ON M5G 2C4, Canada; 8 Medical Intensive Care Unit, Soroka University Medical Center, Ben-Gurion University of the Negev, Beer-Sheva 8410101, Israel

**Keywords:** point-of-care ultrasound, POCUS, pulmonary arterial hypertension, PAH, ambulatory pulmonary hypertension

## Abstract

Pulmonary arterial hypertension (PAH) is a rare condition with the potential to progress to right heart failure. Point-of-Care Ultrasonography (POCUS), used and interpreted in real time at the bedside to further the cardiopulmonary assessment, has the potential to improve the longitudinal care of PAH patients in the ambulatory setting. Patients from PAH clinics at two academic medical centers were randomized to either a POCUS assessment cohort or non-POCUS standard care (ClinicalTrials.gov identifier NCT05332847). The POCUS group received blinded heart, lung, and vascular ultrasound assessments. Thirty-six patients were randomized to the study and followed over time. Mean age was 65 in both groups and majority female (76.5% and 88.9% females in POCUS and control, respectively). Median time for POCUS assessment was 11 min (range 8–16). There were significantly more changes in management in the POCUS group than control (73% vs. 27%, *p*-value < 0.001). Multivariate analysis revealed that management changes were more likely to occur with a POCUS assessment, with an odds ratio (OR) of 12 when POCUS was added to physical exam vs. OR of 4.6 compared to physical examination alone (*p* < 0.001). POCUS in the PAH clinic is feasible and, when combined with physical examination, increases the number of findings and results in changes in management without significantly prolonging visit encounters. POCUS may help support clinical evaluation and decision making in ambulatory PAH clinics.

## 1. Introduction

Pulmonary arterial hypertension (PAH) is an uncommon, progressive, and lethal disease that can eventually lead to right heart failure (RHF) [1]. The pathophysiological changes in the pulmonary vasculature which occur in advanced PAH ultimately lead to functional changes in the right ventricle (RV) [2,3]. An increase in RV afterload eventually results in the disruption of normal ventriculoatrial coupling, pressure and/or volume overload, RV dysfunction, and finally ventricular failure [4,5]. Changes in the RV include wall hypertrophy, chamber dilatation, and bowing of the interventricular septum towards the left ventricle (LV) [6,7]. Point-of-Care Ultrasonography (POCUS) is a rapidly evolving bedside modality in which ultrasonography is utilized as a part of the physical examination and the patients’ clinical assessment and is interpreted in real time at the bedside [8]. During POCUS, the cardiovascular system and the lungs are quickly examined by the clinician, and the information obtained is immediately integrated into the assessment process, therefore directly influencing treatment decisions in the moment [9,10]. Randomized trials and observational data have previously demonstrated the ability of a POCUS assessment to significantly shorten the time to definitive treatment in inpatients with dyspnea [11,12]. Recent data also suggest that individual physical examination findings alone have inadequate diagnostic utility in patients with pulmonary hypertension (PH) [13]. Formal echocardiography, interpreted by a cardiologist, is an essential modality for PAH patient management, including the assessment of disease severity, prognosis, and treatment [3,14,15]. However, a transthoracic echocardiogram (TTE) is not available routinely and expeditiously in an outpatient clinic, official results can be delayed, and obtaining a TTE often necessitates additional contacts with the medical system on the part of the patient. A full formal echocardiogram, if integrated into a clinic visit, extends the duration of the patient visit and is resource-intensive.

POCUS can be used to identify alterations in RV morphology, size, and function, and can provide a more accurate assessment of a patient’s volume status. It has the power to estimate the right ventricular systolic pressure (RVSP) as a surrogate for pulmonary artery pressure, and the presence of a pericardial effusion [11,13]. Furthermore, POCUS provides real-time information on the LV wall size and function, and can establish the diagnosis of valvulopathies [2,9,14,16]. Lung ultrasound can evaluate the presence of an increase in interstitial lung water, pleural effusion, atelectasis, or consolidation [17]. More advanced echocardiographic variables, including the RV/LV basal diameter/area ratio, flattening of the interventricular septum, and the tricuspid annular plane systolic excursion (TAPSE), are all measurable using POCUS at the bedside and are all endorsed in the most recent comprehensive PH European Respiratory Society (ERS) guidelines [18]. These bedside, immediate, additive data may assist clinicians with the identification of PAH progression, complications, and meaningful clinical changes in real time [19], with the potential over a prolonged period of care in the ambulatory setting to shorten time to treatment escalation, hospitalization, or referral to lung transplantation. While thoracic ultrasound has been well studied in the inpatient and intensive care settings and has been shown to change clinical management [20], data extending to the outpatient setting are lacking.

This study sought to evaluate the potential clinical benefits of the addition of a POCUS assessment during an ambulatory PH clinic encounter, conducted by a non-cardiologist as part of the routine clinical patient evaluation, compared to standard clinic practice without ultrasonography. To date, no other study has evaluated the utility of a thoracic cardiopulmonary POCUS assessment in a PH clinic or PAH patient population.

## 2. Materials and Methods

We conducted a randomized controlled pilot study to assess the feasibility and utility of the integration of POCUS into the physical assessment of PH clinic outpatients in two academic centers. This study was registered at ClinicalTrials.gov (ClinicalTrials.gov identifier NCT05332847). Patients were recruited from the PH clinics at the Soroka University Medical Center and Barzilai University Medical Center between January 2018 and March 2019. Patients were included if they were diagnosed with group 1 PAH, as defined by the ERS clinical classification of PH [3], including right heart catheterization, and provided informed consent. Patients were excluded if they were diagnosed with a World Health Organization group 2–5 PH disease, had congenital heart disease, liver cirrhosis, or suspected pulmonary venous occlusive disease.

**Figure 1:**
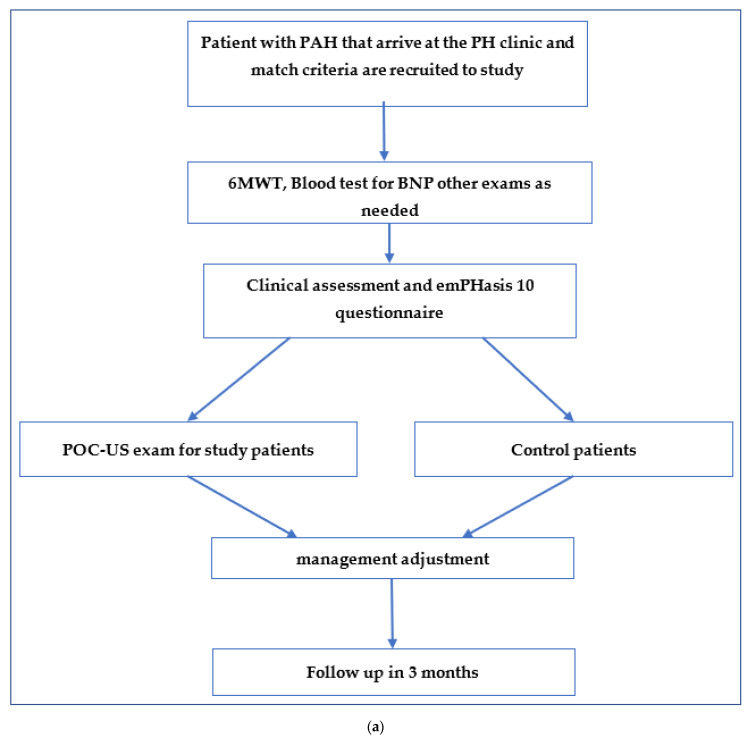

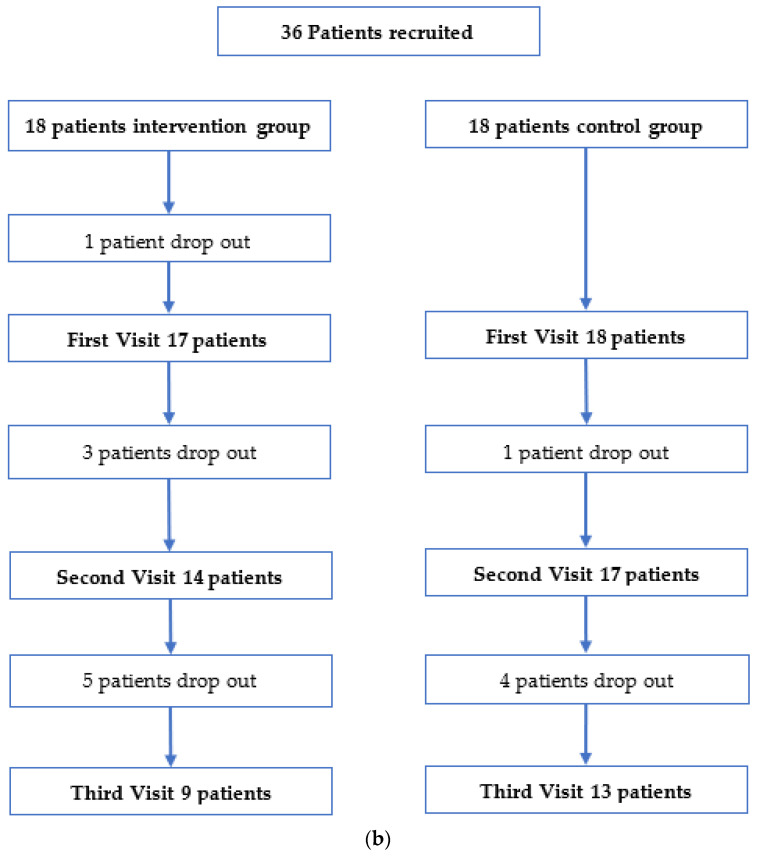
(**a**). Study flow diagram: 6MW—6 min walk test; BNP—brain natriuretic peptide. (**b**). Patient recruitment.

On enrollment, randomization of patients was performed 1:1 (using ClinStat) to either the POCUS assessment group or control group. Both groups underwent the same clinical assessment according to clinic protocols and were evaluated by the same pulmonary hypertension specialist (A.A.) to reduce variability. Every patient was scheduled for a follow-up appointment every three months as part of usual PAH ambulatory clinical care. The study protocol was approved by each participating center’s research ethics board (BRZ 0106-18, SOR0327-16). Clinic visit evaluations for all patients in both groups were standardized (Figure 1a). Each visit included a history, physical and laboratory evaluation, BNP level, 6 min walk test, and a quality-of-life assessment using emPHasis 10, a well-validated health-related quality-of-life questionnaire in PAH [3,21].

All patients in the intervention group underwent a standardized bedside-focused sonographic assessment of the heart, lungs, abdomen, and inferior vena cava (IVC) in every clinic visit during the period of study. The POCUS operator was blinded and not privy to the patients’ medical records, nor any previous assessments, and did not provide any diagnostic or treatment recommendations at any time. The exam was conducted either at the beginning or after the regular patient assessment. Views obtained included 2-dimensional parasternal long and short axis, subcostal long and IVC, apical 5, 4, and 2 chamber views. As this exam was POCUS-centered and not a formal cardiac echocardiographic study, the use of protocolized routine Doppler and size measurements were minimized, and instead a global assessment for chamber function and size was obtained. When specific pathologies were identified, more relevant advanced measurements were taken at the discretion of the scanning POCUS clinician, and the patient was referred for a formal TTE. For example, when a calcified aortic valve was detected, and aortic stenosis was suspected, a continuous pulse-wave Doppler for maximal velocity measurement was conducted. Standard measurements were determined for various indications, including left ventricle wall thickness, chamber size, IVC collapsibility index, RVSP, and size of pericardial effusion when found. TAPSE, which has been shown to correlate with survival in PH [22], was calculated when indicated. Lung ultrasound was focused on the detection of pulmonary congestion (screening and counting B-lines on both lungs, midclavicular line and midaxillary line), pleural effusion, lung atelectasis, lung consolidations (mid and posterior axillary lines), and sliding of pleura for pneumothorax (midclavicular line). Doppler for deep vein thrombosis (DVT) was performed only when suggested by the clinical context.

All POCUS exams were documented in a standardized form (Appendix A) which was made available to the treating pulmonologist in real time and was documented in the medical record (Figure 1a). The treating clinician was not present for the ultrasonographic portion, and the pulmonologist’s exposure to the POCUS case report was the only differing variable between the two study arms. All POCUS studies were performed by a physician (L.F) with six years’ experience in point-of-care ultrasound as a trained operator, with the performance of over 200 heart and lung exams. The POCUS study was designed to take 10 min. Two ultrasound machines were used: GE Vivid S70 with Cardiac Sector Probe M5Sc-D and ESaote Italy/MyLab 5/cardiac probe PA121, Naples, Italy. A cardiac probe, 3 MHZ, was used for the cardiac exam. The same probe was used for the lung ultrasound exam. A linear probe, 9 MHZ, was used for DVT screening when relevant.

The primary outcome was set as change in management to evaluate the association between the POCUS assessment and any changes in clinical management during the ambulatory visit. Management change was defined as any treatment adjustment, including medication changes, hospitalizations, referral for new diagnostic studies or procedures, and other further investigations. We manually counted each management change by reviewing electronic medical records, physician’s notes, and medical orders, and documented the number of changes per visit in each group. Secondary outcomes included six-minute walk test (6MWT) distance, New York Heart Association (NYHA) class, Brain Natriuretic Peptide (BNP) levels, hospitalization rate, ICU admission, quality-of-life assessed by the emPHasis-10 questionnaire, and the length of the clinic visit as measured from arrival to departure from clinic. POCUS evaluation, lab results, six-minute walk test, and medical decisions were documented in the Case Report Form (Appendix B) and the patients’ medical record.

Baseline characteristics of the study population were examined across allocated intervention groups. Data are reported as percentages for categorical variables and means + standard deviations or medians (interquartile ranges) for continuous variables, according to the variable’s distribution. Data were compared across intervention groups, at baseline and follow-up, using Chi-square statistical test for categorical variables and Mann–Whitney or independent T-test for continuous variables, according to the variable’s distribution. To further assess for the primary outcome, multivariate logistic regression was performed to control for potential confounders and other possible influential variables. The multivariable models also specifically included the possible interaction between abnormal physical examination and abnormal POCUS examination as two dichotomous variables. The physical examination in the model was marked as normal or abnormal when one or more of the following was detected: increased jugular vein distention, peripheral or pulmonary edema, hepatomegaly, or ascites. In the statistical model, the abnormal physical exam was marked as “yes” or “no” and was entered to the logistic regression model as such. Convenient sample size for a pilot feasibility study was set to thirty patients. Significance levels were set to a two-sided *p*-value of <0.05. Statistical tests were performed using SPSS v. 26 (IBM, Armonk, NY, USA).

## 3. Results

In this 13-month prospective study period, we enrolled 36 patients from 2 academic PH clinics. Eighteen were randomized to the intervention group and eighteen to the control group. Twenty-two patients from both groups completed three follow-up visits each (Figure 1b). Two patients declined to participate in the study and the remaining patients that dropped out of the study did so due to the physical distance of their homes from the PAH clinic. Table 1 summarizes demographic and clinical characteristics at enrollment. There was no crossover of patients between study groups. Demographics and health status was similar between groups. At enrollment, the intervention POCUS group had a lower median NYHA score (NYHA class 2 (1–3) compared to NYHA class 3 (2–4) in the control group, *p* = 0.05) (Table 1). Despite this, 6-MWT, BNP measurements, signs of RV failure, and EmPHasis-10 scores were similar between cohorts. The median time for POCUS assessment was 11 min (range 8–16 min) across ambulatory visits in the intervention arm, however there was no significant difference between the median total visit time between the intervention and control arm (29 vs. 30 min, *p* = 0.105).

**Figure 2:**
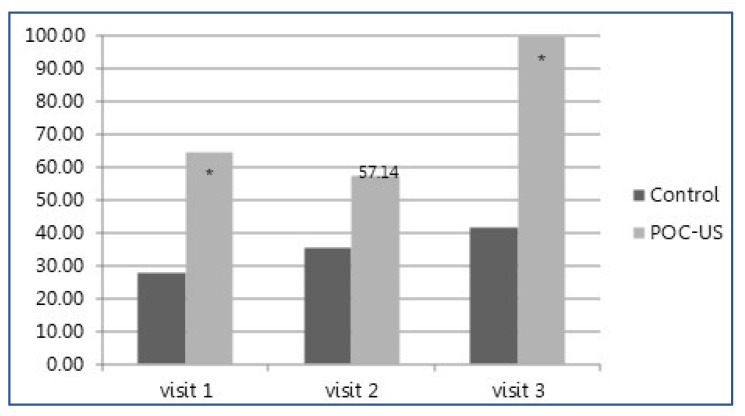
Management change rate per visit. Management change by treatment group presented as percentage of patients exposed to a change. * refers to a statistically significant difference, *p* value < 0.05.

More management changes overall, as well as more total absolute number of visits with management changes, occurred in the POCUS group compared to the control (48 (73%) vs. 18 (27%) *p* < 0.001, and 32 (74.4%) vs. 17 (34.7%), respectively, *p* < 0.001) (Table 2). The total number of changes in clinical management on a per-visit basis was significantly higher in the POCUS intervention group (1.2 vs. 0.37 *p* < 0.001, Table 2). Figure 2 shows the number of changes for each of the three clinic visits, shown as a percentage of total patients exposed to any management change. For all documented visits across the duration of the study follow-up, the number of changes was significantly higher in the POCUS group (visit 1, 64.71% vs. 27.78 %; visit 2, 57.14 % vs. 35.29%; and visit 3, 100 % vs. 41.67%, respectively). Across the study period, of the 17 patients in the intervention group, 14 (82%) had any change in management, while in the control group, 11 out of 18 patients (61%) had similar changes recorded. In the POCUS group, across 43 total outpatient visits, 33% of encounters included a medication change (*n* = 14), while 72% included a diagnostic change (*n* = 31), a statistically significant difference compared to in the control group, where only 17.6% of visits included a medication-related change and 15.7% of visits included a diagnostic change (*p* value = 0.094 for medication changes and 0.005 for non-medication-related changes).

In the multivariable regression models, the POCUS intervention study arm and the abnormal physical examination were the only variables that were statistically significant for influencing management change (Table 3). In the fully adjusted multivariable model, POCUS remained significantly associated with management change with an OR of 11.98 (3.59–40.01, *p* < 0.001), while abnormal physical examination had an OR of 4.66 (1.34–16.22, *p*-value = 0.01) (Table 3). There was no significant interaction between physical examination abnormality and POCUS intervention (*p* > 0.1). The inclusion of an abnormal physical examination to the model increased the association of POCUS and management changes dramatically.

Analysis of secondary outcomes revealed no significant differences between study groups. Hospitalization rate (6% in POCUS group vs. 4.7% in control, *p*-value 0.79), new PH-specific medication prescriptions (28% in POCUS group vs. 32.6% in the control group, *p*-value 0.778), ICU admission rate (2% in the POCUS group vs. 0 in the control group, *p*-value 1), EmPHasis-10 score (23% in the POCUS group vs. 25% in the control group, *p* value = 0.93), and PH-specific therapy medication changes (2% in the POCUS group vs. 4.8% in the control group, *p*-value 0.398) were all statistically unchanged between the two cohorts (Appendix C).

Table 4 summarizes 26 of the relevant significant POCUS findings in our study. The most common findings were “unexpected normal” RVSP/central venous pressure (CVP), where elevation had been anticipated, and LV hypertrophy. Table 5 presents case series examples of some POCUS findings from the intervention arm and the management changes and interventions that followed.

## 4. Discussion

We present, for the first time in a pilot randomized control trial, that the integration of POCUS as an adjunct to the physical examination of the heart and lungs during an outpatient visit in patients with PAH in the ambulatory setting is feasible, rapid, and leads to significantly more management changes and treatment adjustments compared to the standard patient assessment.

Technological advances have improved the portability and lowered the price of ultrasound systems, bringing this modality to the patients’ bedside in the ambulatory clinic. POCUS has infiltrated almost all hospital wards, from the emergency departments to the intensive care units [8]. POCUS training has been incorporated into medical educational curricula, with the standardization of teaching, at the novice level, how to integrate this modality into daily practice across disciplines [23]. Previous studies have shown that POCUS can be used as a tool to affect decision making in the clinical setting and improve patient management and time to definitive treatment [11,12,24,25]. Recently, the international multi-center Ultra Man study showed that the use of thoracic ultrasound at the bedside in an inpatient population led to frequent changes in clinical impressions and subsequent meaningful management changes in nearly half of the patients evaluated with POCUS [20]. POCUS has been less utilized outside the hospital in ambulatory environments, with minimal reports of POCUS assessment in the pre-hospital setting [26,27,28], and few descriptions of utility in outpatient pulmonary hypertension ambulatory clinics. Samant et al. described the use of portable handheld ultrasound to measure right atrial pressures in an outpatient PH clinic and demonstrated a correlation with BNP and clinical worsening [29].

Our study looked to examine the feasibility and impact of introducing a comprehensive cardiopulmonary POCUS assessment into a PAH clinic as an additional tool for making quick, effective, and potentially cost-effective therapeutic decisions. We demonstrated in a blinded, randomized fashion that POCUS assessment is feasible in the ambulatory PH setting and has the potential to significantly and meaningfully affect patient care, with an increase in total management changes and in the frequency of such changes, as compared to traditional care sans POCUS. These changes encompassed both meaningful diagnostic and medication-related alterations in care. Other downstream clinical outcomes including rate of hospitalization due to PAH exacerbations, referral to lung transplantation, and death during the study period did not differ between groups, likely as a result of the small sample size and short interval follow-up time. This study was designed to serve as a feasibility trial to demonstrate proof of concept and serve as a foundation for future investigations and was not designed or powered to determine changes in these potentially more meaningful clinical outcomes.

In an additional multivariate analysis, our findings were reinforced with the demonstration that the only independent factors associated directly with PAH management changes were the findings in both the physical examination and POCUS (Table 3). The statistical significance of the relationship between POCUS and clinical management changes was even stronger when physical examination was combined with POCUS. We did not find any management changes independently associated with BNP, 6 min walk, or EmPHasis-10 questionnaire (Table 3). Examples of POCUS findings that influenced management are described in Table 5. For example, POCUS assessment of Patient 12 during visit three revealed pulmonary B lines, indicating pulmonary edema. In response to this, the pulmonologist added a diuretic drug and ordered a formal TTE, which would not have occurred without the additional clinical information POCUS provided. In another example, POCUS of Patient 23 diagnosed a high CVP, an elevated RVSP, and a restrictive diastolic pattern on ultrasound, prompting the addition of a diuretic and a formal re-evaluation of cardiac and LV function. Patient 24 on visit three was diagnosed with LVH and multiple B lines reflecting lung congestion, and in response to these findings, the treating physician ordered a new right heart catheterization.

Our results demonstrate meaningful clinical changes when outpatient PH clinics incorporate POCUS assessment into the routine patient evaluation. The integration of this ultrasound modality in the clinic is feasible to upgrade the physical exam, known to perform poorly in PH [13], in the diagnosis of pathology that could not be found by the conventional examination (Table 4 and Table 5). This clinical benefit came without an increase in the total time of the outpatient clinic encounter, as the POCUS assessments took only 10 min to perform in a comprehensive manner. POCUS assessment is viable in the outpatient clinic and has potential in the hands of a trainer operator to decrease time to appropriate treatment, and potentially more meaningful downstream clinical outcomes.

This study was designed as a hypothesis-generating feasibility study and was not powered to evaluate clinical outcomes such as improved functional status or survival, nor whether they were related to the management changes recorded. To assess these clinical outcomes and causality, a prospective controlled study with a larger enrollment and longer follow-up is needed. Our study serves as the foundation and suggestion for this future work. In addition, our study did not have a protocolized treatment algorithm that was based on the POCUS findings. Rather, it was designed to serve as a real-world surrogate, where the clinician reacted to the POCUS findings using their own clinical judgment. Only one treating pulmonologist and one trained POCUS operator were involved in the study, reducing the risk of inter-operatory variability, albeit potentially limiting the generalizability of the findings. However, with the advent of POCUS across medical disciplines and the growth of critical care echocardiography [30], these skills are expanding beyond the first wave of highly trained operators into routine clinical practice. In future studies, the feasibility of pulmonary hypertension specialists in performing their own POCUS studies should be evaluated. In this regard, the training of physicians to conduct and become proficient in POCUS also needs to be considered and prioritized. While this may take time in an ambulatory clinic, brief training sessions have been shown to meaningfully improve the skills of novice operators in both the short and long term [31,32] and there continues to be significant interest in ultrasound education across medical training [33,34]. Hopefully, our results support the notion that this time investment is worthwhile, with the strong potential to be meaningfully beneficial to patient care. It is our position, supported by the literature, that the real-time POCUS assessment is important to patients’ management by making new diagnoses that prompt management changes, shortening time to definitive treatment [11,12,20,24,35]. Our pilot study results support and hopefully will serve to inform the conduct of a larger multicenter prospective trial to evaluate the role of POCUS in improving patient outcomes in the ambulatory setting in patients with PH.

## 5. Conclusions

POCUS integrated into the physical examination during routine PAH clinic encounters increases the number and rate of management changes compared to standard usual care without ultrasonography and is feasible without sacrificing ambulatory clinic time in patient visits. POCUS is a valid diagnostic tool that may help the treating physician at the bedside and support a clinical cardiopulmonary evaluation in patients with pulmonary hypertension in the outpatient environment. Further, prospective, larger studies are needed to confirm if POCUS assessment in this setting affects downstream clinical patient outcomes.

## Figures and Tables

**Table 1:** Baseline characteristics.

Variable		POCUS (*n* = 17)	Control (*n* = 18)	*p*-Value
Sex (female) *n* (%)		13 (76.5%)	16 (88.9%)	0.402
Age (years) mean ± SD		65 ± 14	65 ± 16	0.934
BMI (weight) mean ± SD		29 (±6.3)	28.7 ± 10.9	0.666
Duration of disease (month) median (IQR)		33 (8, 39)	21 (5, 30)	0.105
Left ventricular dysfunction *n* (%)		2 (11.8%)	0	0.229
Lung disease *n* (%)		2 (11.8%)	0	0.402
Chronic kidney disease *n* (%)		1 (5.9%)	1 (5.6%)	1
Chronic liver disease *n* (%)		1 (5.9%)	0	0.486
Malignancy *n* (%) PAH Diagnosis:		1 (5.9%)	1 (5.6%)	1
Connective tissue disease		2 (11.8%)	7 (38.9%)	0.121
IPAH		14 (82.4%)	9 (50%)	0.044
FPAH		0	2 (11.1%)	0.486
Drug and toxins exposure		1 (5.9%)	0	0.486
HIV		0	0	NA
PH medications	No	2 (11.8%)	3 (16.7%)	0.194
	Yes	7 (41.2%)	11 (61.1%)	0.862
	>1 PH drug	8 (47.1%)	4 (22.2%)	0.862
Diuretics		8 (47.1%)	9 (50%)	0.862
NYHA Class	1	1(5.9%)	0	0.05
	2	8 (47.1%)	4 (22.2%)	0.05
	3	8 (47.1%)	14 (77.8%)	0.05
Symptoms on enrolment	None	6 (35.3%)	5 (27.8%)	0.948
	Orthopnea	1 (5.9%)	0	0.948
	Chest pain	3 (17.6%)	0	0.948
	Palpitations	0	0	0.948
	Syncope	0	0	0.948
	Edema	1 (5.9%)	2 (11.1%)	0.948
	Increased abdominal girth	0	0	0.948
	Constitutional symptoms	0	0	0.948
	Dyspnea	6 (35.3%)	11 (61.1%)	0.948
Right heart failure		5 (31.3%)	7 (38.9%)	0.642
BNP mean ± SD		758 ± 1420.3	847 ± 1064.3	0.369
6 Min walk test mean ± SD		374 ± 166	334 ± 98	0.179
EmPHasis-10 evaluation median (IQR)		19 (8, 26)	26 (18, 33)	0.143

Abbreviations: BMI, body mass index; CTD, connective tissue disease; IPAH, idiopathic pulmonary artery hypertension; FPAH, familial pulmonary arterial hypertension; HIV, human immunodeficiency virus; NYHA, New York Heart Association; BNP, brain natriuretic peptide.

**Table 2:** Total management changes.

Variable	POCUS	Control	*p* Value
Total PH outpatient visits	43	49	1
Visits with management changes (%)	32 (74.4%)	17 (34.7%)	<0.001
Total management changes	48	18	<0.001
Average management changes per visit	1.2	0.37	<0.001

**Table 3:** Models for management change rate factors among two study cohorts.

Model	Odds Ratio	Confidence Interval 95%		*p*-Value
		Lower	Upper	
Model 1				
Age	0.99	0.96	1.02	0.51
Sex (male)	1.7	0.45	6.3	0.43
POCUS group	6.02	2.3	15.9	<0.001
Model 2				
Age	0.99	0.96	1.02	0.47
Sex (male)	1.8	0.45	7.03	0.40
Symptoms	1.12	0.96	1.30	0.14
BMI	0.99	0.94	1.04	0.99
POCUS group	8.7	2.92	25.97	<0.001
Model 3				
Age	0.98	0.95	1.01	0.23
Sex (male)	2.34	0.56	9.89	0.24
Symptoms	1.17	0.96	1.30	0.16
BMI	0.97	0.92	1.03	0.42
Physical examination	4.66	1.34	16.22	0.01
POCUS group	11.98	3.59	40.02	<0.001

**Table 4:** POCUS findings.

POCUS Findings	Number (%) Total *n* = 26
Suspected aortic stenosis	1 (3.8)
Right ventricular hypertrophy	1 (3.8)
Left ventricular hypertrophy	5 (19.3)
“Unexpectedly normal” RVSP	6 (23.2)
Pericardial effusion	1 (3.8)
Pulmonary congestion	4 (15.4)
D-shaped septum	2 (7.7)
Arrhythmia	1 (3.8)
Elevated central venous pressure	3 (11.5)
Restrictive diastolic pattern	2 (7.7)

**Table 5:** Selected examples of management changes following POCUS findings.

Patient Code/Visit	POCUS Findings	Management Change
Patient 1/Visit 2	Calcified aortic valve Suspected aortic stenosis	A new formal TTE study was ordered
Patient 10/Visit 1	Signs of LVH, small pericardial effusion B-lines suggestive of pulmonary congestion	Diuretics were added
Patient 12/Visit 3	B lines suggestive of pulmonary congestion	Diuretics were added A new formal Echo study was ordered
Patient 21/Visit 1	Surprisingly normal POCUS study with low CVP	Diuretics were stopped A new right catheterization was scheduled
Patient 22/Visit 1	Small left ventricle Low CVP	Diuretics dosage was reduced Chest computed tomography study ordered Full pulmonary function test ordered A new right catheterization was scheduled
Patient 23/Visit 2	High CVP Elevated RVSP (58 mm Hg) Restrictive diastolic pattern	A new formal TTE to assess suspected diastolic left ventricular dysfunction was ordered
Patient 24/visit 2	B lines suggestive of lung congestion	Diuretic dosage increased
Patient 24/visit 3	LVH B lines suggestive of lung congestion	A new right catheterization was scheduled
Patient 30/visit 1	Elevated CVP Elevated RVSP (55) D-shaped Septum	A new formal TTE study was ordered

## Data Availability

The data presented in this study are available on request from the corresponding author. The data are not publicly available due to privacy restrictions.

## Appendix C. Other Secondary Outcomes

**NYHA (Mean ± SD)** **[median]**	**POCUS**	**Control**	***p*-Value**
Visit 1	2.4 (±0.6) [2]	2.8 (±0.4) [3]	0.05
Visit 2	2.1 (±0.6) [2]	2.8 (±0.5) [3]	0.012
Visit 3	2.4 (±0.7) [3]	2.9 (±0.3) [3]	0.05
**6 MWT (meter)** **(Mean ± SD) [median]**	**POCUS**	**Control**	***p*-Value**
Visit 1	396 (±134) [423]	334 (±98) [333]	0.084
Visit 2	373 (±171) [433]	373 (±95) [361]	0.184
Visit 3	334 (±165) [371]	353 (±77) [364]	0.8
**BNP (Mean ± SD) [median]**	**POCUS**	**Control**	***p*-Value**
Visit 1	757.9 (±1420) [141]	690 (±979) [285]	0.495
Visit 2	734.5 (±1373) [158]	517 (±1049) [147]	0.817
Visit 3	519 (±873) [257]	730 (±1180) [273]	0.849
**emPHasis-10** **(Mean ± SD) [median]**	**POCUS**	**Control**	***p*-Value**
Visit 1	20.8 (±12.8) [24]	25.5 (±11) [23]	0.248
Visit 2	23.9 (±13.4) [28]	23.7 (±8.4) [24]	0.878
Visit 3	30.3 (±15) [34]	24.1 (±10) [24.5]	0.227
**Events of worsening**	**POCUS**	**Control**	***p*-Value**
Visit 1	-	-	-
Visit 2	2 (14.3%)	4 (23.5%)	0.664
Visit 3	1 (11.1%)	2 (16.7%)	1
**Prostaglandin** **initiation *n* (%)**	**POCUS**	**Control**	***p*-Value**
Visit 1	1 (5.9%)	0	0.486
Visit 2	0	0	-
Visit 3	0	0	-

**Transplantation** **referral *n* (%)**	**POCUS**	**Control**	***p*-Value**
Visit 1	1 (5.9%)	1 (5.6%)	1
Visit 2	0	1 (5.9%)	1
Visit 3	1 (11.1%)	0	0.429
**Add-on PH medication *n* (%)**	**POCUS**	**Control**	***p*-Value**
Visit 1	5 (29.4%)	6 (33.3%)	1
Visit 2	5 (35.7%)	5 (29.4%)	1
Visit 3	4 (44.4%)	3 (25%)	0.397
**Medication switch *n* (%)**	**POCUS**	**Control**	***p*-Value**
Visit 1	2 (11.8%)	0	0.229
Visit 2	0	0	-
Visit 3	0	1 (8.3%)	1
**Management change *n* (%)**	**POCUS**	**Control**	***p*-Value**
Visit 1	11 (64.7%)	5 (27.8%)	0.044
Visit 2	8 (57.1%)	6 (35.3%)	0.289
Visit 3	9 (100%)	5 (41.7%)	0.003

**NYHA change *n* (%) visit 2**	**POCUS, *n* = 14 (%)**	**Control, *n* = 17 (%)**	***p*-Value**
increased	1 (7.1%)	4 (23.5%)	0.428
decreased	5 (35.7%)	4 (23.5%)	
**NYHA change *n* (%) visit 3**	**POCUS, *n* = 9 (%)**	**Control, *n* = 13 (%)**	***p*-Value**
increased	2 (22.2%)	4 (36.4%)	0.79
decreased	1 (11.1%)	1 (9.1%)	
